# Predominantly defective CD8^+^ T cell immunity to SARS-CoV-2 mRNA vaccination in lung transplant recipients

**DOI:** 10.1186/s12967-023-04234-z

**Published:** 2023-06-08

**Authors:** Ellie Taus, Michael Y. Shino, F. Javier Ibarrondo, Mary Ann Hausner, Christian Hofmann, Otto O. Yang

**Affiliations:** 1 Department of Molecular and Medical Pharmacology, David Geffen School of Medicine, University of California, Los Angeles, Los Angeles, CA 90095 USA; 2 Department of Medicine, David Geffen School of Medicine, University of California Los Angeles, Los Angeles, CA 90095 USA

**Keywords:** SARS-CoV-2, COVID-19 mRNA vaccine, Solid organ transplantation, Lung transplantation, Cellular immunity

## Abstract

**Background:**

Although mRNA vaccines have overall efficacy preventing morbidity/mortality from SARS-CoV-2 infection, immunocompromised persons remain at risk. Antibodies mostly prevent early symptomatic infection, but cellular immunity, particularly the virus-specific CD8^+^ T cell response, is protective against disease. Defects in T cell responses to vaccination have not been well characterized in immunocompromised hosts; persons with lung transplantation are particularly vulnerable to vaccine failure with severe illness.

**Methods:**

Comparison groups included persons with lung transplantation and no history of COVID-19 (21 and 19 persons after initial mRNA vaccination and a third booster vaccination respectively), 8 lung transplantation participants recovered from COVID-19, and 22 non-immunocompromised healthy control individuals after initial mRNA vaccination (without history of COVID-19). Anti-spike T cell responses were assayed by stimulating peripheral blood mononuclear cells (PBMCs) with pooled small overlapping peptides spanning the SARS-CoV-2 spike protein, followed by intracellular cytokine staining (ICS) and flow cytometry for release of cytokines in response to stimulation, including negative controls (no peptide stimulation) and positive controls (phorbol myristate acetate [PMA] and ionomycin stimulation). To evaluate for low frequency memory responses, PBMCs were cultured in the presence of the mRNA-1273 vaccine for 14 days before this evaluation.

**Results:**

Ionophore stimulation of PBMCs revealed a less inflammatory milieu in terms of interleukin (IL)-2, IL-4, and IL-10 profiling in lung transplantation individuals, reflecting the effect of immunosuppressive treatments. Similar to what we previously reported in healthy vaccinees, spike-specific responses in lung transplantation recipients were undetectable (< 0.01%) when tested 2 weeks after vaccination or later, but were detectable after in vitro culture of PBMCs with mRNA-1273 vaccine to enrich memory T cell responses. This was also seen in COVID-19-recovered lung transplantation recipients. Comparison of their enriched memory responses to controls revealed relatively similar CD4^+^ T cell memory, but markedly reduced CD8^+^ T cell memory both after primary vaccination or a booster dose. These responses were not correlated to age or time after transplantation. The vaccine-induced CD4^+^ and CD8^+^ responses correlated well in the healthy control group, but poorly in the transplantation groups.

**Conclusions:**

These results reveal a specific defect in CD8^+^ T cells, which have key roles both in transplanted organ rejection but also antiviral effector responses. Overcoming this defect will require strategies to enhance vaccine immunogenicity in immunocompromised persons.

**Supplementary Information:**

The online version contains supplementary material available at 10.1186/s12967-023-04234-z.

## Background

Since the emergence of SARS-CoV-2 as a human pathogen in 2019, it rapidly spread to cause a pandemic responsible for millions of deaths. Fortunately, the rapid deployment of mRNA vaccines encoding the viral spike protein has dramatically reduced the mortality from COVID-19. While vaccine-generated antibody responses are important for preventing initial symptomatic infections, cellular immune responses have the major role in preventing morbidity and mortality [1–4].

Epidemiologic studies have shown that persons who are immunosuppressed due to solid organ transplantation have increased risk for severe disease and death from COVID-19, with the greatest risk associated with lung transplantation [5–8]. This is presumably related to the relatively higher degree of immunosuppression after lung transplantation compared to other common organ transplantations (kidney, heart, liver), as well as the lung involvement in COVID-19. Of concern, the mRNA vaccines have demonstrated reduced efficacy in solid organ transplantation patients, including lung transplantation patients [9, 10].

Many studies have demonstrated poor antibody responses to mRNA SARS-CoV-2 vaccination in persons after solid organ transplantation, with overall seroconversion rates of only about 34% and 66% after initial vaccination and a third booster vaccination respectively (recently reviewed in [11]), but detailed information about cellular immune responses is relatively limited despite their importance in protecting from severe illness. Here we evaluate vaccine-elicited cellular immunity against spike in lung transplant recipients.

## Methods

### Study approval

All work was performed under an institutional review board-approved protocol at the University of California Los Angeles. Prior to participation, all subjects gave written informed consent.

### Participants and samples

The lung transplantation and healthy control vaccine evaluation participants had no known history of COVID-19. The healthy control individuals had no immunocompromising medical conditions and had negative antibodies against the SARS-CoV-2 spike receptor-binding domain (RBD) by ELISA at baseline before vaccination. The COVID-19-recovered lung transplantation individuals had PCR-documented infection during hospitalization. PBMC were separated by ficoll density gradient centrifugation and viably cryopreserved until use. All samples were taken prior to winter of 2021, when primary vaccinations and initial booster vaccinations had first been implemented.

### Intracellular cytokine staining (ICS) to detect T cells targeting spike

ICS staining and flow cytometry were performed as described in detail [12], except differing in the antigenic target. In brief, PBMCs were incubated with a pool of overlapping 15-mer peptides spanning spike [13] at a final concentration of 1 µg/ml of each individual peptide, with brefeldin A (#00-4506-51, eBioscience, San Diego, CA) and monensin (#00-4505-51, eBioscience, San Diego, CA), followed by surface staining CD3-Super Bright 436, CD8-Super Bright 600, CD4 PE-Cy7, and Fixable Aqua viability dye (#62-0037-42, eBioscience, San Diego/CA; #63-0088-42, eBioscience, San Diego/CA; #25-0049-42, San Diego, CA; and #L34957, Invitrogen, Waltham, MA respectively), permeabilization (#00-5523-00, eBioscience, San Diego, CA), and intracellular cytokine staining for interferon (IFN)-ɣ-FITC, IL-2-PerCP-Cy5.5, IL-4-PE, and IL-10-APC (#506504 Biolegend, San Diego, CA; #500322, Biolegend, San Diego, CA; # 130-091-647, Miltenyi Biotec, Bergisch Gladbach, Germany; and #506807, Biolegend, San Diego, CA respectively) for flow cytometric analysis.

### In vitro enrichment of memory T cells against spike

In parallel with ICS evaluation for anti-spike T cell responses immediately upon thawing, a portion of the PBMC was cultured with the mRNA-1273 vaccine in vitro. One to two million PBMC per well were maintained in 24-well flat bottom tissue culture plates in RPMI 1640 (supplemented with l-glutamine, HEPES buffer, and antibiotic) with recombinant human IL-2 at 50 U/ml (NIH AIDS Reagent Repository Program) and the initial addition of mRNA-1273 vaccine (Moderna) at 1 ng/ml. The cultures were repleted with fresh medium every three to 4 days. The resulting cells were utilized for ICS evaluation of anti-spike T cell responses after approximately 14 days of culture. Aliquots were viably cryopreserved; if ICS staining yielded fewer than 10,000 events each in the CD4^+^ or CD8^+^ T cell compartments, the ICS was repeated on another aliquot and the results were combined with weighted averaging.

### Statistics

Comparisons of group means were performed using Student’s t-test or Fisher’s exact test for continuous and binary variables, respectively. Comparisons of group Kaplan–Meier type memory frequency curves were performed using log-rank tests. Evaluations for correlations were performed with Spearman’s rank tests.

## Results

### Profile of lung transplantation recipients and non-immunosuppressed control participants

Basic clinical information is given for the lung transplantation recipients and healthy control subjects in Table 1 and the Additional file 2. Two groups of transplantation participants without a history of COVID-19 were studied as a group of 21 who had been primarily vaccinated but not yet boosted (two doses of mRNA vaccine, “vaccinated”) and a group 19 who were vaccinated and boosted with a third dose of mRNA vaccine (“boosted”). 18 of the 19 boosted subjects were longitudinally evaluated from the initial vaccinated group (one individual was not tested before the booster dose). A third smaller group of transplantation participants had recovered from documented COVID-19; all of these were vaccinated or vaccinated and boosted at the time of sampling; vaccinations occurred before or after COVID-19. On average, the lung transplantation groups were somewhat older than the control group (mean age 62 vs 65 vs 52 years), included more females (48% vs 42% vs 71%), and had less use of the mRNA-1273 vaccine (100% vs 100%/95% vs 27%). Most of the lung transplantation recipients were on a stable regimen of prednisone, tacrolimus, and mycophenolate per the UCLA lung transplantation program at the time of vaccination, with a few receiving sirolimus or azathioprine. All samples were obtained prior to winter of 2021. Except for the COVID-19 group, all persons had no history of COVID-19. The control subjects were seronegative for antibodies against the spike RBD just before vaccination. The lung transplantation subjects were not tested before vaccination, but their antibody levels after primary vaccination and/or booster vaccination were typical for SARS-CoV-2-naïve solid organ transplantation subjects (the majority were seronegative, and seropositive persons had low titers, not shown).

**Table 1:** Subject group characteristics

Group		Number	Mean age yrs (SD)	Female/male	Lungs transplanted double/single	On sirolimus	On tacrolimus	On prednisone	On MMF	On azathioprine	Initial vaccine mRNA-1273/BNT162b2	Booster vaccine mRNA-1273/BNT162b2	Mean years from TX to first VAC	Mean years from TX to COVID-19	Mean days from VAC to test (SD)	Mean days from boost to test (SD)	Mean days from COVID onset to test (SD)
A	Transplant vaccinated (unboosted)	21	61.8 (10.6)	10/11	11/10	5 (24%)	20 (95%)	21 (100%)	13 (62%)	2 (10%)	21/0	N/A	4.5 (3.2)	N/A	99.4 (43.8)	N/A	N/A
B	Transplant boosted (3rd dose)	19	64.5 (9.5)	8/11	8/11	4 (21%)	19 (100%)	19 (100%	11 (58%)	2 (11%)	19/0	18/1	4.6 (3.2)	N/A	189.4 (21.5)	17.1 (10.9)	N/A
C	Transplant covid-19^*^	8	58.9 (12.9)	3/5	5/3	0 (0%)	8 (100%)	8 (100%)	8 (100%)	0 (0%)	8/0^**^	4/0^***^	2.2 (1.1)	2.4 (1.1)	165.6 (85.4)	53.0 (28.4)^**^	134.4 (147.2)
D	Healthy controls vaccinated (unboosted)	21	51.9 (10.6)	15/6	N/A	N/A	N/A	N/A	N/A	N/A	6/16	N/A	N/A	N/A	121.0 (50.4)	N/A	N/A
Statistical comparisons																	
A vs D			0.0037	0.2082							< 0.0001				0.1417		
B vs D			0.0003	0.1086											< 0.0001^****^		
A vs B			0.4017	0.7605	0.5450	1.0000	1.0000	1.0000	1.0000	1.0000			0.9719		< 0.0001^****^		

General characteristics are compared between groups of lung transplantation subjects and healthy control subjects. Relevant statistical analyses for differences (p values by Student’s t-test or Fisher’s exact test as appropriate) are given for comparisons between the groups. The post-COVID-19 group was not included in statistical comparisons due to its small number

^*^3/8 were tested after COVID-19 occurred after vaccination with a third booster dose, 2/8 were tested after vaccination (no boost) after recovery from COVID-19, 2/8 were tested after COVID-19 occurred after vaccination (no boost), 1/8 was tested after vaccination and a third booster vaccination after COVID-19

^**^All eight persons were vaccinated either before or after COVID-19

^***^Includes only the four persons who received a booster vaccination at some time before testing

^****^Comparison of days from most recent vaccination (either primary vaccination or third booster vaccination)

### The general cytokine profiles of T cells in lung transplant patients show differences from controls, suggesting a less immunoreactive milieu

**Fig. 1:**
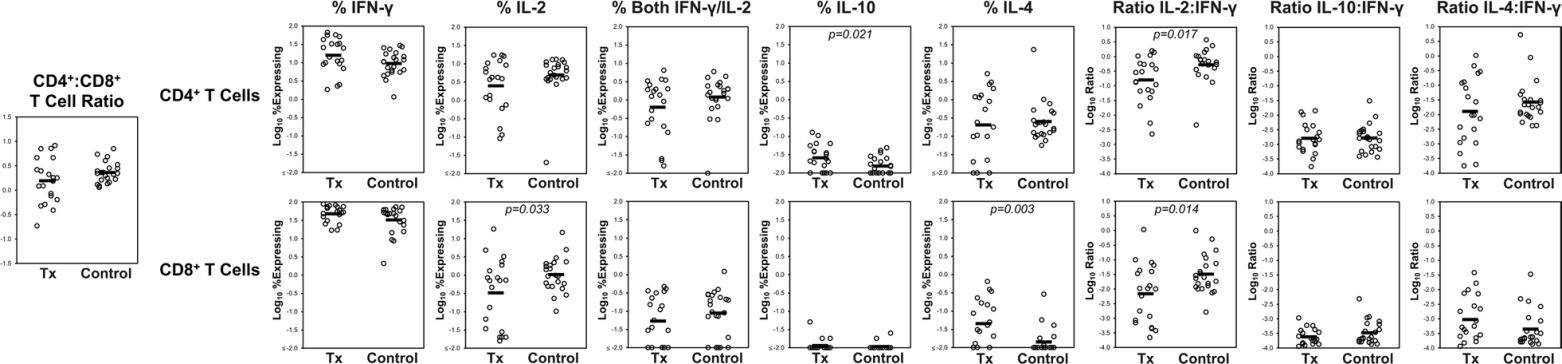
Global cytokine profiling of peripheral blood T cells comparing lung transplantion recipients to healthy control individuals. Cytokine profiles were assessed in 20 individuals with lung transplantation (Tx) and 22 healthy non-immunocompromised (Control) individuals. PBMC were stimulated with PMA/ionomycin and evaluated for production of IFN-γ, IL-2, IL-10, and IL-4 by CD4^+^ and CD8^+^ T cells by flow cytometry. Comparison plots between subject groups are shown for ratio of CD4^+^:CD8^+^ T cells, percentages of cells producing IFN-γ, IL-2, both IFN-γ and IL-2, IL-10, IL-4, and ratios of IL-2:IFN-γ, IL-10:IFN-γ, and IL-4:IFN-γ are shown. Comparisons with statistically significant differences (p < 0.05 by Student t-test) are indicated. All persons had received two doses of COVID-19 mRNA vaccination (without booster vaccination); the transplanted group ranged from 14 to 177 days (mean 83) after vaccination, and the control group ranged 21 to 235 days (mean 127 days) after vaccination

PBMCs from 22 healthy control individuals and 20 lung transplantation recipients were evaluated for global production of interferon-gamma (IFN-γ), interleukin-2 (IL-2), interleukin-10 (IL-10), and interleukin-4 (IL-4) after PMA/ionomycin stimulation (Additional file 1: Figure S1, Fig. 1, Additional file 2). The ratios of CD4^+^ and CD8^+^ T cells were relatively similar between these groups. Comparing the CD4^+^ T cell compartments, on average there were higher percentages of IL-10-producing cells and lower ratios of IL-2:IFN-γ production in the transplant group. Between the CD8^+^ T cell compartments, on average there were higher percentages of IL-4-producing cells and again lower ratios of IL-2:IFN-γ production in the transplant group. The increase in the immunosuppressive cytokine IL-10 in CD4^+^ T cells and the increase in the Th2-biased cytokine IL-4 in CD8^+^ T cells, with the reduced balance of IL-2 production in both subsets, was consistent with an environment of reduced cellular immunoreactivity. For antigen-specific measurements, only IFN-γ responses were reliably detected (Additional file 2), and the remainder of the analyses focused on these responses.

### In lung transplant recipients, spike-specific T cells after initial COVID-19 vaccination and boosting are mostly undetectable in circulation soon after vaccination, but robust responses are seen for most persons after in vitro vaccine stimulation to enrich memory responses

**Fig. 2:**
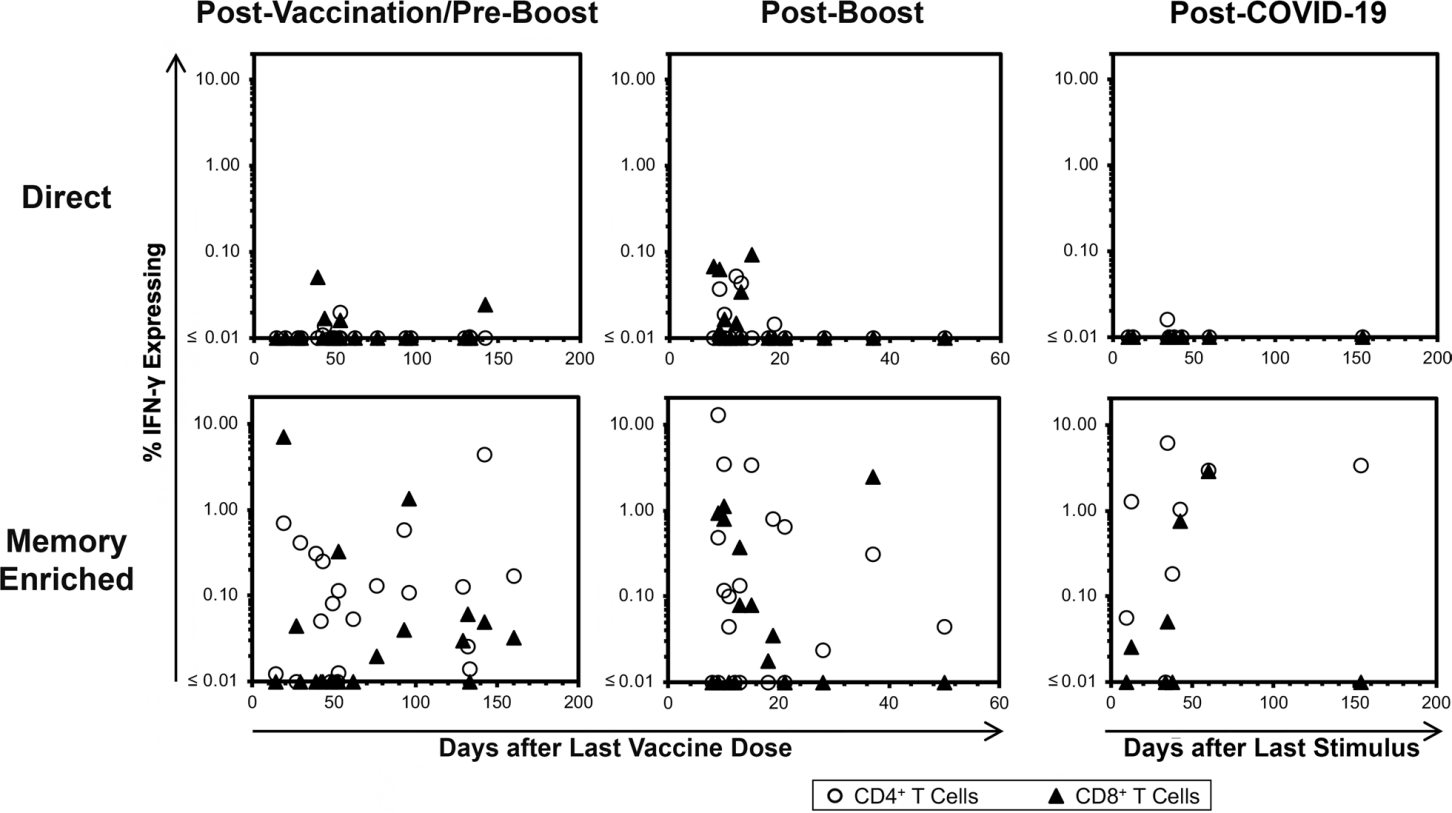
Minimal detection of spike-specific T cell responses after COVID-19 mRNA vaccine administration or COVID-19 infection, but readily detectable responses after enrichment by PBMC culture with mRNA-1273 vaccine in persons after lung transplantation. Spike-specific T cells in blood of lung transplant recipients were assayed by intracellular cytokine staining for IFN-γ production by flow cytometry after exposure to pooled peptides spanning the spike protein. 21 COVID-19-naïve persons were evaluated after completion of primary vaccination with two doses of mRNA COVID-19 vaccines without a third booster dose (range 42 to 160 days after second dose, mean 71 days, first column), 19 COVID-19-naïve persons were evaluated after primary vaccination and a third “booster” vaccination dose (range 8 to 50 days after booster vaccination, mean 17 days, second column), and 8 persons who had had COVID-19 (with or without vaccination, third column) were evaluated

PBMC from lung transplantation recipients were evaluated for spike-specific T cell responses by flow cytometric detection of IFN-γ production (Additional file 1: Figure S1) after exposure to pooled small peptides spanning spike (Fig. 2, Additional file 2). Evaluations were performed in 21 lung transplantion recipients without a history of COVID-19 who completed the two initial doses of vaccination but had not yet received a third “booster” dose, and 19 who had received the third booster dose. Additionally, 8 lung transplantation recipients who had a history of COVID-19 (either before or after vaccination) were also evaluated. In all three situations (Fig. 2 top row), most persons had no detectable responses, and most of the detected responses were low frequency and observed soon after vaccination. When the same PBMC samples were cultured with the addition of mRNA-1273 vaccine for approximately 2 weeks before assessment for spike-specific T cell responses (Fig. 2 bottom row), responses were readily detectable for most persons. In the CD4^+^ T cell compartment 18/21 (86%), 13/19 (68%), and 7/8 (88%) of persons had detectable memory (> 0.01%) after initial vaccination, booster vaccination, or COVID-19 respectively. There were fewer responders in the CD8^+^ T cell compartment, where 10/21 (48%), 9/19 (47%), and 4/8 (50%) of persons in these groups had detectable memory, respectively. Within the time frame of these measurements, there was no apparent time-dependence in these cross-sectional examinations (Fig. 2). Relevant to comparing the lung transplantation and healthy control vaccine groups (Table 1), the latter was younger in age and more likely to have received the BNT162b2 vaccine. Also, sampling was performed sooner in the boosted transplantation group than the other transplantation vaccination group or the control group. There were too few subjects in the COVID-19 transplantation group to make meaningful comparisons. Overall, however, these findings were consistent with observations of similarly short-lived responses after mRNA vaccination of non-transplanted individuals that could be enriched to reveal memory responses after in vitro culture with mRNA − 1273 vaccine [14].

### Compared to healthy control individuals, persons with lung transplantation have similar levels of vaccine-elicited CD4^+^ T cell responses after mRNA COVID-19 vaccination, but reduced CD8^+^ T cell responses even after a third booster vaccination

**Fig. 3:**
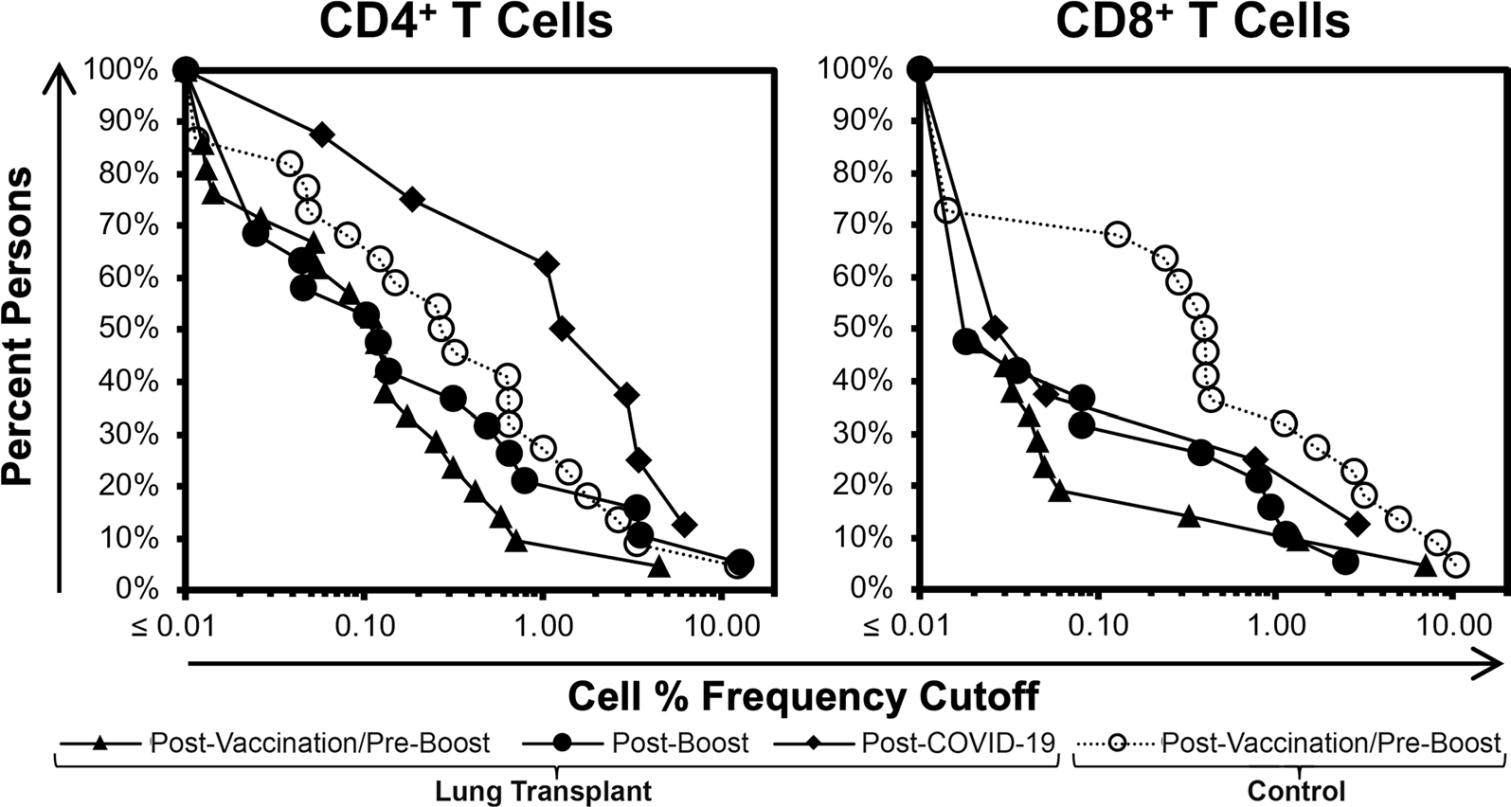
Comparison of in vitro-enriched memory responses against spike after mRNA vaccination elicited by vaccination or COVID-19 in persons with lung transplantation versus vaccinated healthy control persons demonstrates a defect predominately in the CD8^+^ T cell subset. Boosted memory T cell responses against spike were assayed (as per Fig. 2) in 21 lung transplantation individuals who had received two doses of primary vaccination but no booster vaccination (tested 42 to 188 days after vaccination, mean 99 days), 19 lung transplantation individuals who had subsequently received booster vaccination (tested 8 to 50 days after booster vaccination, mean 17 days), 8 lung transplantation individuals who had recovered from COVID-19 that occurred either before or after vaccination and/or booster vaccination, and 22 healthy control non-transplanted individuals who had received only two doses of primary vaccination (tested 21 to 235 days after vaccination, mean 121 days). For the CD4^+^ (left) and CD8^+^ (right) T cell memory responses, plots are given for the percentages of persons (Y-axis) who had detected spike-specific enriched memory responses at or above certain frequencies (X-axis). Log-rank test statistical comparisons in the CD4^+^ subsets revealed no significant differences between control versus transplant-vaccinated, control versus transplant-boosted, and transplant-vaccinated versus transplant-boosted (p-values of 0.091, 0.84, and 0.57 respectively). In the CD8^+^ subsets, comparisons between control versus transplant-vaccinated, control versus transplant-boosted, and transplant-vaccinated versus transplant-boosted (p-values of 0.00075, 0.022, and 0.15) demonstrated that the control group memory responses were significantly higher than both transplant groups. Statistical comparisons to the COVID-19-recovered group were not performed due to the small number of subjects

Vaccine-induced memory T cell responses against spike were also evaluated with the in vitro mRNA-1273 culture assay using PBMCs from healthy non-immunosuppressed control persons (who had been vaccinated but not yet boosted) for detailed quantitative comparisons (Fig. 3 and Additional file 2). In these controls, CD4^+^ and CD8^+^ memory T cell responses were detectable above 0.01% in 19/22 (86%) and 16/22 (73%) of persons, respectively (without booster vaccination). Comparing these enriched memory responses to those elicited by vaccination in the lung transplantation group, the frequencies of detected CD4^+^ T cell memory were statistically similar between control individuals and both groups of transplant individuals after vaccination with or without boosting, although there was a statistically non-significant trend for higher levels in the latter (Fig. 3 left). However, in the CD8^+^ T cell subset, the frequencies in both transplantation groups (before or after booster vaccination) were significantly lower than the control individuals, again with statistically non-significant slightly higher responses in the boosted vaccination group (Fig. 3 right). In the few evaluated COVID-19 transplantation subjects, CD4^+^ T cell responses appeared higher than controls, while CD8^+^ T cell responses appeared similarly low to the two vaccinated transplantation groups (Fig. 3). Again, there were significant differences in age, BNT162b2 versus mRNA-1273 vaccination rate, and sampling time between groups. Overall, however, these results suggested that the frequencies of vaccine-elicited spike-specific memory was relatively normal for CD4^+^ T cells, but reduced for CD8^+^ T cells in persons with lung transplantation.

### For the ranges of the vaccination groups, neither age nor time after lung transplantation appeared to have influence on levels of enriched memory responses

**Fig. 4:**
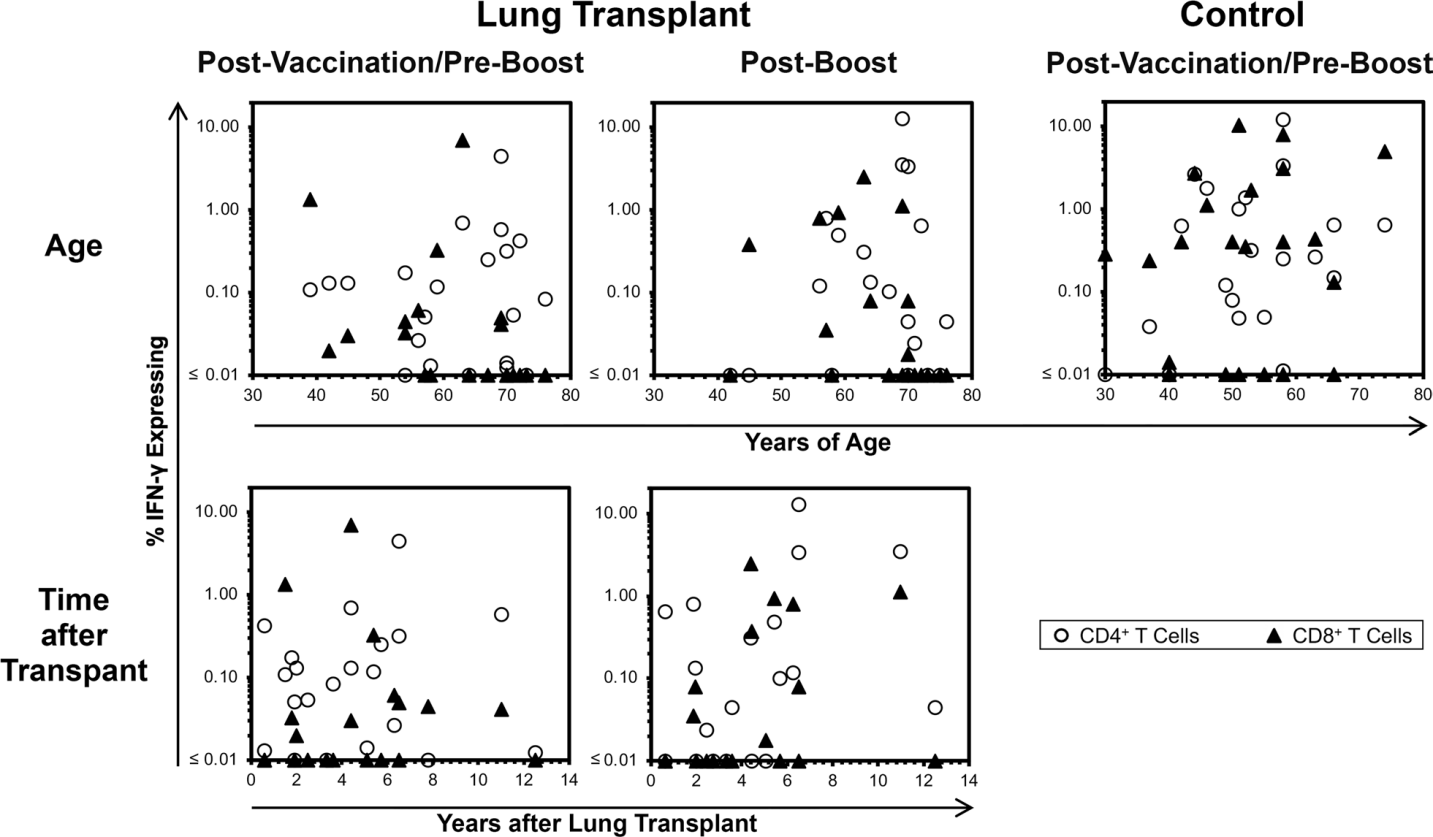
Evaluation for correlations of boosted memory responses to age or time after lung transplantation demonstrates no clear relationships. The enriched memory response measurements from Figs. 2, 3 were plotted against years of age (top row) or years after lung transplantation (bottom row)

To evaluate whether the detection of enriched memory responses was biased by age or time after lung transplantation in these subjects, the responses evaluated in Figs. 2, 3 were replotted against these two parameters (Fig. 4). Across all three groups, enriched CD4^+^ and CD8^+^ T cell response magnitudes demonstrated no association with age. Within the two lung transplantation groups, there also was no evidence association with time since transplantation with CD4^+^ and CD8^+^ T cell responses. These results suggested that for the span of ages and times since lung transplantation, these parameters were not significant determinants of vaccine response.

### The magnitudes of enriched CD4^+^ and CD8^+^ T cell vaccine responses correlated in healthy control persons but not persons after lung transplantation

**Fig. 5:**
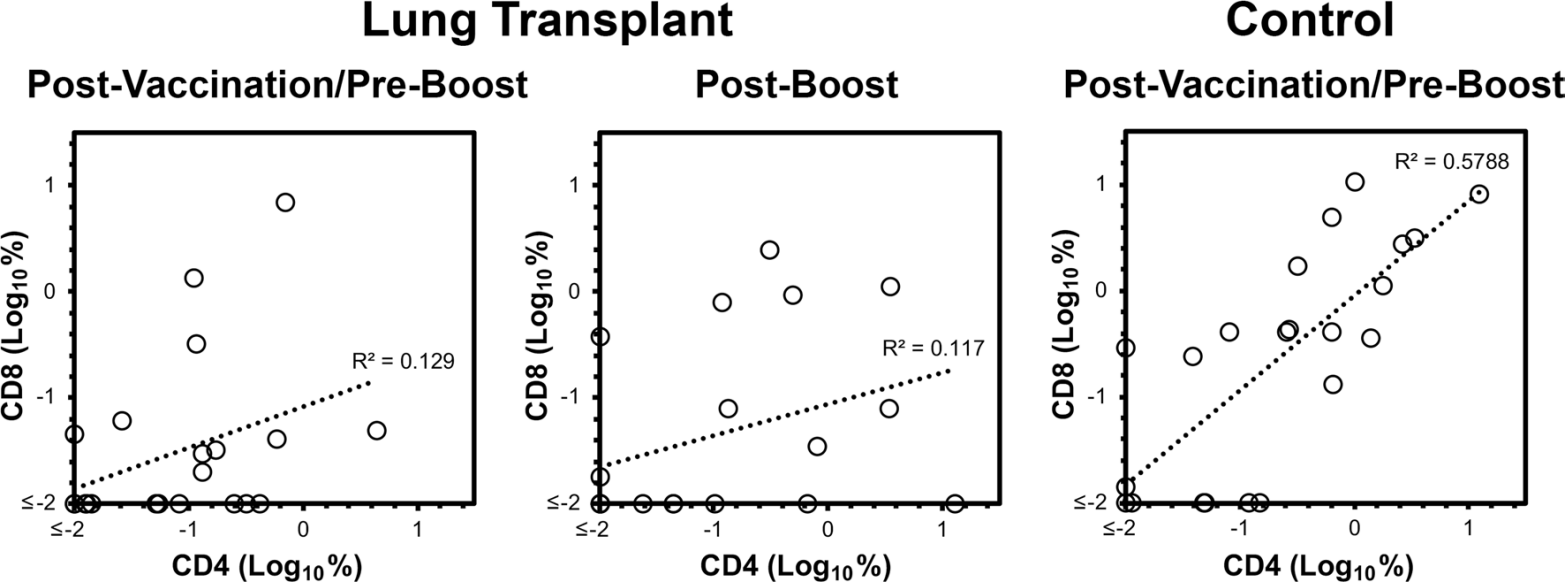
Enriched CD4^+^ and CD8^+^ T cell responses to vaccination correspond to each other in healthy control persons but not lung transplantation recipients. The enriched memory T cell responses to vaccination from Figs. 2, 3 were analyzed for correlation of the CD4^+^ and CD8^+^ T cell subsets. By Spearman rank test, the lung transplantation group correlations were not significant (p = 0.12 and p = 0.40), while the correlation in the healthy control group was highly significant (p < 0.0001)

To further examine the degree to which enriched memory T cell responses were depressed generally versus specifically in the CD8^+^ T cell subset in lung transplantation, the CD4^+^ and CD8^+^ responses were compared for each group of subjects (Fig. 5). Correlations were poor in both lung transplantation groups, while the correlation was robust in the healthy control group. These results supported a biased defect of the CD8^+^ T cell response in persons with lung transplantation.

## Discussion

Given the particular vulnerability of persons with solid organ transplantation to severe COVID-19 and their reduced protection by vaccines, there has been interest in understanding the immune defects underlying these issues. Given relatively accessible methodologies to measure antibodies, many studies have focused on humoral immune responses after vaccination [15–22], and these studies have consistently demonstrated markedly diminished antibody responses compared to non-immunocompromised individuals [11].

Due to technical challenges of measuring cellular immune responses, it has been more difficult to define defects of T cell responses to vaccination in transplantation patients. In non-immunocompromised persons, we previously showed that T cell responses peak rapidly about 7 to 10 days after each SARS-CoV-2 mRNA vaccine dose and then fall to undetectable levels by ELISpot or intracellular cytokine staining for IFN-γ by about 20 days [23]. This apparent peculiarity of mRNA vaccines yields a very narrow window to directly detect T cell responses from vaccination.

Of the studies of vaccination in solid organ transplantation subjects, to our knowledge only one study, Sattler et al. evaluated cellular immunity in this window of peak responses after vaccination [24]. They evaluated persons with renal transplantation about 8 days after completing BNT-162b2 vaccination, using flow cytometry for T cell activation to find that 92% of subjects had detectable CD4^+^ T cell responses (but with significantly lower magnitude than healthy controls) while only 5% had detectable CD8^+^ T cell responses. This study therefore yielded results consistent with our findings of better CD4^+^ T cell responsiveness compared to CD8^+^ T cells. Whether this pattern holds after contraction of the T cell response to the later memory phase, however, has been unclear.

Other groups have attempted to measure T cell responses later after vaccination with varying success and results. Yanis et al. evaluated solid organ transplant recipients 21–42 days after completing vaccination with BNT162b2 by flow cytometry using activation markers, and detected only borderline responses in CD4^+^ T cells and none in CD8^+^ T cells [25]. In agreement with our findings, Hall et al. evaluated solid organ transplant subjects about 5 weeks after vaccination, using intracellular cytokine staining for IFN-γ and IL-2, finding that 23/48 and 1/48 persons had CD4^+^ and CD8^+^ T cell responses respectively [26]. The reason for the discrepancy between their being able to detect responses so late after vaccination compared to our negative findings with straight PBMCs is unclear, but could be related to methodologic differences (they used co-stimulatory antibodies against CD28 and CD49d, and performed a longer peptide incubation). Havlin et al. evaluated 12 lung transplant recipients about 9 weeks after BNT162b2 vaccination by intracellular cytokine staining flow cytometry and found 4/12 had both CD4^+^ and CD8^+^ T cell responses [27]. Their methodologic capacity to detect responses this late after vaccination is uncertain; they used only 1 to 1.5 million PBMCs and described responses as low as 0.005% of CD4^+^ or CD8^+^ T cells, and transplant recipients are typically lymphopenic. In a further study using the same methodology, they assessed 15 lung transplant recipients about 3 months after BNT162b2 vaccination/pre-boost and then about 3 weeks after a third booster dose [21]. They observed no difference in the number of CD4^+^ and CD8^+^ T cell responders either pre-boost (1/15 and 1/15 persons) or post-boost (4/15 and 4/15 persons).

Our findings stand apart from these prior studies because we are able to assess memory T cell responses robustly after enrichment by PBMC culture with the mRNA-1273 vaccine, after contraction from their initial peak and decay of frequencies below detection limits in standard assays [23]. This allows a clear examination and comparison of cellular immunity targeting spike in lung transplant recipients versus non-immunocompromised persons. The data provide a clear demonstration of a predominant defect in the CD8^+^ T cell compartment, extending the observations immediately after vaccination of Sattler et al. [24] to weeks after these initial responses have decayed, and confirming the later trends seen less clearly by Yanis et al.[25] and Hall et al. [26].

A caveat is that our methodology to measure memory T cell responses is semi-quantitative because it relies on cell expansion in vitro. The final detected level of memory T cells depends both on initial frequency as well as proliferative capacity that is subject to variability in culture. However, parallel comparisons of the lung transplant recipients and controls reveal clear differences in the CD8^+^ T cell compartment, and assay variability would serve to reduce the power to see differences between these groups. Similar methodology using stimulation with peptides [28–31] or live vaccinia vaccine [32, 33] to enhance detection of low frequency memory responses has also been used in several vaccine studies. This methodology does not distinguish whether observed difference in the CD8^+^ T cell compartment is due to lower starting frequency and/or reduced proliferative capacity in the lung transplant recipients, and whether the difference predicts less vaccine protection. However, frequency and proliferative capacity are closely related and there is likely a deficit of both in transplant recipients. Antiviral function is also interrelated with these T cell properties, and it is also likely that our results at least indirectly reflect vaccine protection from severe illness in COVID-19.

The other key caveat regards the characteristics of the participant groups. There were significant differences in several factors that affect comparisons of responses between the vaccine groups. Ages were significantly different between the lung transplantation and control individuals, the latter being younger on average by about 10 years. We cannot exclude that this was a factor in the greater vaccine responsiveness of the control group, although it seems unlikely that there would be a dramatic difference in that age range, and evaluation of age as a factor in the control group (who ranged from ages 30 to 74 years) found no significant influence of age. Another significant difference was the predominant vaccination of the transplantation group with mRNA-1273 versus BNT162b2 vaccination of the control group. However, these vaccines are very similar in design and identical in mechanism, and have been shown to be similarly immunogenic for cellular responses in healthy persons [34]. The other key factor was timing of sampling after vaccination. While the vaccinated but unboosted control and transplantation groups were similar in sampling time after vaccination and therefore comparable, the boosted transplantation group was sampled much earlier after vaccination. Since later sampling would bias for lower responses, it is therefore unclear if the slightly higher (but statistically non-significant) response after boosting was due to timing of sampling or due to increase by the third booster dose. In either case, the responses of the boosted group were clearly lower than the control group, since the bias would favor the opposite of what was observed. Overall, we cannot exclude that the age bias could play a minor role in our findings, but the vaccine formulation and sampling time differences would bias against our observations. Our findings may in fact underestimate the defect in CD8^+^ T cells, given the longer time from vaccination in the controls (mean 121 days) compared to transplant recipients (99 or 17 days) at the time of evaluation. Although the cross-sectional comparison of memory responses showed no clear trend for decline, it is known that T cell responses elicited by COVID-19 [12, 35] and SARS-CoV-2 vaccines [36–38] decay over months, consistent with waning of vaccine protection from severe illness [39–43]. This underscores the generally critical role of cytotoxic CD8^+^ T cells clearing viral infections through recognition of infected cells.

Additionally, we evaluated a small number of lung transplantation recipients who survived COVID-19. While there were too few participants to make firm conclusions and statistical comparisons, this group showed very similar trends to the vaccination groups (preserved CD4^+^ T cell responses and defective CD8^+^ T cell responses) compared to the controls. This was consistent with impaired immunity even in the setting of the greater antigenic challenge of natural infection compared to vaccination.

The cause of reduced vaccine responsiveness after solid organ transplantation is not clear. Several studies have implicated treatment with mycophenolate in reduced humoral immune responses to mRNA SARS-CoV-2 vaccines [17, 19, 25] and breakthrough infection after vaccination [44]. For the reasons discussed above, most studies of T cell responses to vaccination of solid organ transplantation recipients have not provided clear measurements, and thus the role of specific immunosuppressive treatments on cellular immunity has not been delineated. In our study, most lung transplant recipients were on similar treatment regimens (all on prednisone and tacrolimus and/or sirolimus, and most on mycophenolate or azathioprine) and thus the contributions of individual agents could not be determined. Because T cells play a central role in transplanted organ rejection [45, 46], and cytotoxic CD8^+^ T cells comprise the major effector arm of cellular immunity, it is perhaps not surprising that the immunosuppressive regimens effective in preventing rejection also especially blunt the protective antiviral CD8^+^ T cell response.

Although we did not find a statistically significant increase in cellular immune memory after a third booster dose compared to initial completion of vaccination, it has been observed clinically that solid organ transplant recipients have increased protection from severe disease [10]. This suggests that there may be improved cellular immunity after the booster, and perhaps the small increase we observed was functionally relevant and not due to the age bias discussed above. Alternatively, lack of an observed increase could be explained by inadequate quantitative precision of our memory T cell expansion assay. Another possibility is that the clinical benefit of a third booster dose could be due to the contribution of antibodies that can increase after a poor response to primary vaccination [47], and which do not correlate to the cellular immune response [27].

## Conclusions

In summary, we find that most lung transplantation recipients do have persisting memory T cell responses against spike after primary vaccination with mRNA vaccines against SARS-CoV-2 despite their rapid decay to levels below the limits of detection in standard assays. However, compared to non-immunocompromised persons, these individuals have a marked deficit in memory CD8^+^ T cell responses. This defect is not appreciably reduced after a third “booster” vaccination. Given the critical role of CD8^+^ T cells both in clearing viral infections and causing rejection of transplanted organs, this highlights the significant challenge posed by immunosuppressive treatments to prevent rejection. Whether reduced responsiveness to the mRNA vaccines can be corrected by increased dosage or frequency of vaccination should be explored, given now-established endemicity of SARS-CoV-2 in the human population.

## Supplementary Information

**Additional file 1: Figure S1** Example of flow cytometric assessment of T cell cytokine production. An example is shown for PBMC stimulated with PMA and ionomycin. Single T cells were identified by gating via forward/side scatter, dead cell exclusion, CD3, and CD4 or CD8. Intracellular cytokine staining revealed production of IFN-γ, IL-1, IL4, and IL-10 in CD4 and CD8 T cell subpopulations.

**Additional file 2.** Supplemental raw data spreadsheet.

## Data Availability

All raw data are provided as a Additional file 2.

## Abbreviations

PBMC: Peripheral blood mononuclear cells
ICS: Intracellular cytokine staining
PMA: Phorbol myristate acetate
IL: Interleukin
IFN: Interferon
RBD: Spike receptor-binding domain
MMF: Mycophenolate mofetil
Tx: Transplant
