# Supplementary figures and images for: Predominantly defective CD8+ T cell immunity to SARS-CoV-2 mRNA vaccination in lung transplant recipients
Source: J Transl Med. 2023 Jun 8;21:374. doi: 10.1186/s12967-023-04234-z (PMC10248978; doi:10.1186/s12967-023-04234-z)

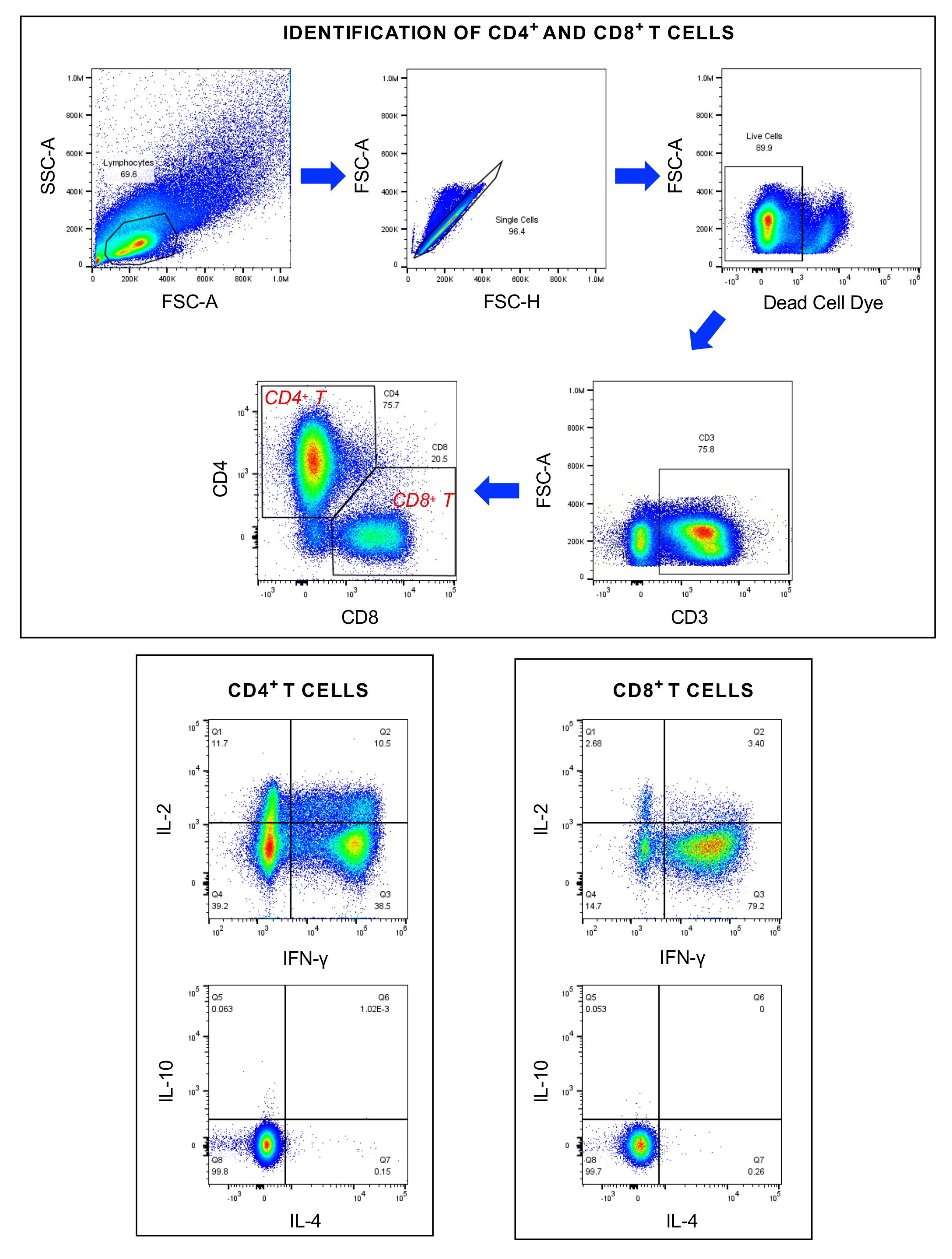

Supplement: Supplementary file 1 — Additional file 1: Figure S1 Example of flow cytometric assessment of T cell cytokine production. An example is shown for PBMC stimulated with PMA and ionomycin. Single T cells were identified by gating via forward/side scatter, dead cell exclusion, CD3, and CD4 or CD8. Intracellular cytokine staining revealed production of IFN-γ, IL-1, IL4, and IL-10 in CD4 and CD8 T cell subpopulations. [file 12967_2023_4234_MOESM1_ESM.tif]
